# FUNCTIONAL MRI TO PREDICT RECOVERY FROM DISORDERS OF CONSCIOUSNESS AFTER BRAIN INJURY

**DOI:** 10.1093/geroni/igad104.3126

**Published:** 2023-12-21

**Authors:** Bianca Rodriguez, Christopher Boivin, David Fischer

**Affiliations:** Hospital of the University of Pennsylvania, Philadelphia, Pennsylvania, United States; Hospital of the University of Pennsylvania, Philadelphia, Pennsylvania, United States; Hospital of the University of Pennsylvania, Philadelphia, Pennsylvania, United States

## Abstract

Disorders of consciousness (DoC) following brain injury are challenging for families, who often decide about end-of-life care without a clear understanding of a patient’s recovery. This has special significance for older and geriatric populations, as they often rely on caregivers, proxies, and family members in making health and life decisions, and now face the added severity of decisions surrounding continuing treatment. In improving neuroprognostication, we hope to decrease this burden in aging-related decision-making. Functional magnetic resonance imaging (fMRI) has proved helpful in identifying brain activity in DoC with implications for recovery of consciousness, function, and quality of life. This project seeks to develop and implement a standardized fMRI protocol to best induce signals of consciousness in participants and predict the likelihood of neurologic recovery. From there, we will measure how the protocol contributes to an accurate outcome prediction following a DoC as compared to standard methods. The study will be conducted in the intensive care unit(s) of the Hospital of the University of Pennsylvania; we anticipate having an initial analysis of results by Fall of 2023. The study will be enrolling and conducting fMRI’s with the device over one year under a randomized control trial. This study emphasizes improvements in the practice of neuroprognostication after stroke, anoxic brain injury following cardiac arrest, and other related conditions. The literature on these particular forms of traumatic brain injury and neuroprognostication remains limited, which is of disservice to older and geriatric populations who suffer these events at higher rates.

